# Behavioural mechanisms underlying parasite-mediated competition for refuges in a coral reef fish

**DOI:** 10.1038/s41598-019-52005-y

**Published:** 2019-10-29

**Authors:** Graham E. Forrester, Erin Chille, Katie Nickles, Kiran Reed

**Affiliations:** 1 University of Rhode Island, Department of Natural Resources Science, Kingston, 02881 USA; 2 University of Rhode Island, Department of Biological Science, Kingston, 02881 USA

**Keywords:** Food webs, Behavioural ecology

## Abstract

Parasites have been increasingly recognized as participants in indirect ecological interactions, including those mediated by parasite-induced changes to host behaviour (trait-mediated indirect interactions or TMIIs). In most documented examples, host behaviours altered by parasites increase susceptibility to predation because the predator is also a host (host-manipulation). Here, we test for a TMII in which a parasitic copepod modifies the predator-prey interaction between a small goby host and several larger predatory fish. Gobies compete for crevices in the reef to avoid predation and goby mortality increases more rapidly with increasing refuge shortage for parasitized gobies than for those free of parasites. We found interactive effects of refuge shortage and parasitism on two behaviours we predicted might be associated with parasite-mediated competition for refuges. First, as refuge-shortage increases, the rate of aggression among gobies increases and parasitism intensifies this interaction. Second, goby proximity to refuges increases as refuges become scarce, but parasitism nullifies this increase. In combination, these parasite-induced changes in behaviour may explain why parasitized gobies are poor competitors for refuges. Because the parasite is not trophically transmitted via host manipulation, these altered behaviours in parasitized gobies are likely coincidental to infection.

## Introduction

Indirect species-interactions, in which pair-wise interactions between species are modulated by a third, and sometimes fourth, species have long been recognized for their potentially important influence on community dynamics^1,2^. Before these influences on multi-species communities can be explored^3,4^, the types of indirect interactions occurring in these small groups must be identified. Although not represented in some early classifications^1,2^, parasites have been increasingly recognized as participants in indirect interactions, primarily as mediators of predatory and competitive interactions^5–8^.

**Figure 1:**
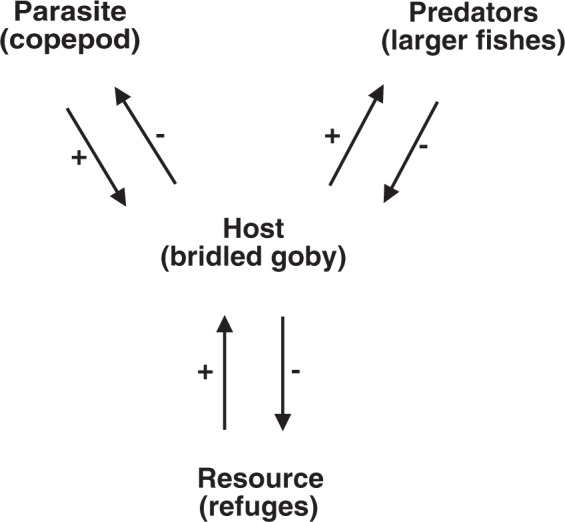
The indirect interaction between gobies, parasites and predators. The host (bridled goby) is infected by a parasite (the copepod *Pharodes tortugensis*), and is also consumed by predators (several larger species of fish). Gobies compete for structural refuges (crevices in the reef) to avoid being consumed by predators, and the parasite reduces their effectiveness as competitors.

Indirect effects of parasites can include density-mediated effects as well as trait-mediated indirect effects (TMIIs sensu^9^). Density mediated host-parasite interactions begin with parasite-induced reduction in host abundance^6^, whereas TMIIs are triggered by parasite-induced changes in host behaviour or phenotype^7,8^. Parasites can alter host behaviour in numerous ways, and the potential for parasite-induced changes in host behavior to affect a host’s susceptibility to predators and/or its competitive ability has long been recognized^5,10,11^. Most research thus far on this topic has focused on cases where parasites manipulate the behaviour of prey intermediate hosts in ways that increase parasite transmission to a predator that is also the final host^12,13^. More recently, however, an increasing variety of other potential indirect interactions that include host-parasite relationships has been recognized and organized into new classifications of TMIIs^6–8^. Although some of these indirect interactions have been carefully studied, there are still relatively few concrete examples because the experimental and observational approaches routinely used to identify predator-prey TMIIs^14^ are less commonly applied to host-parasite interactions^6^. Here, we test for a trait-mediated interaction in which parasites modify a predator-prey interaction (sensu^6^). Although some examples of parasite-mediated predator-prey interactions have been identified^6^, this interaction differs from others reported because the prey/host competes for refuges to avoid predation (Fig. 1).

In this study, the host species, the bridled goby (*Coryphoptererus glaucofraenum* Gill) is infected by a parasitic copepod (*Pharodes tortugensis* Wilson) that attaches to its gills. Gobies infected with *P. tortugensis* grew 66% slower, had 68% smaller gonads, and died at almost twice the rate of uninfected gobies^15,16^. Although parasites may kill some goby hosts directly, gobies are also consumed by several larger species of reef fish, and predator exclusion shows that predation is the most important proximate agent of mortality^17,18^. Vulnerability to predation is mediated by the fact that, when threatened by predators, gobies flee rapidly to take shelter inside reef crevices. Crevices are used only temporarily and are not guarded by gobies. When refuges become locally scarce relative to the number of gobies, the scramble for refuges resembles the childhood game of musical chairs, where gobies compete intraspecifically for the limited refuges^19,20^. Of several plausible measures of crowding, prey mortality is best predicted by a simple measure of refuge shortage: the ratio of gobies to refuges^20^.

Of these past findings, the most suggestive of a potential TMII is that the progressive increase in goby mortality with increasing refuge shortage is more severe for parasitized gobies than for those free of parasites. This finding raises the possibility that parasitic infection reduces gobies’ effectiveness as competitors for refuges^21^. Collectively, this past work suggests this interaction between gobies, predators, and refuges may represent a novel indirect interaction, but parasite-mediated changes in host behaviour or phenotype consistent with a TMII have not been previously explored.

We tested whether behavioural interactions associated with competition for refuges are modified by the parasite and so represent a TMII. We made observations of infected and uninfected hosts at differing levels of refuge shortage. We hypothesized that behaviours associated with a parasite-mediated TMII would change with refuge shortage, and that the degree of change would be amplified or diminished by parasitism. Because the hosts interact with one another at relatively low rates^15,19,21^, we compiled behavioural observations made during four past studies in which goby density and refuge availability were either manipulated or observed at varying levels to create a gradient of refuge shortage (Table 1). We tested predictions about (1) the rate and outcome of aggressive interactions, and (2) the proximity to refuges and area covered while foraging, because theory and past work indicate that these might be associated with competition for refuges and/or modified by parasitic infection (Table 2)^15,19,21^. We also tested the prediction that feeding rates are not associated with refuge shortage and modified by parasitic infection (Table 2), because we hypothesized that this parasite-mediated TMII did not involve competition for food^21^.

**Table 1:** Summary of the four studies from which behavioural observations of focal gobies were compiled for the analysis.

Study	Year	Observations of uninfected gobies (n)	Observations of infected gobies (n)	Study design	Replicates	Habitat matrix
1	2000	73	24	Manipulation of goby and refuge density	4 × 4 m plots (n = 16)	Large area of continuous reef
2	2001	34	38	Observations in plots that varied naturally in goby and refuge density	4 × 4 m plots (n = 20)	Large area of continuous reef
3	2016	44	11	Manipulation of goby and refuge density	4 × 4 m plots (n = 16)	Large area of continuous reef
4	2018	267	90	Manipulation of goby density and parasite prevalence	2 × 2 m patch reefs (n = 12)	Sandy bay

See methods for further details of the procedures used in each study.

**Table 2:** Predicted changes in goby behaviour associated with a parasite-mediated TMII.

Prediction		Basis for prediction
1	The rate of aggressive interactions increases with refuge shortage and the increase is amplified by parasitism	Increased time engaged in aggressive interactions may be associated with “jockeying” for position near refuges and/or may compromise vigilance and reaction times when predators approach
2	The likelihood of losing an aggressive encounter increases with refuge shortage and the increase is amplified by parasitism	Losing in aggressive interactions may be associated with unsuccessfully “jockeying” for position near refuges
3	Distance from a refuge declines with increased refuge shortage and the decline is lessened by parasitism	The chance of successful flight to a refuge when threatened or attacked by a predator should increase for gobies who spend more time closer to a refuge
4	Movement increases with increased refuge shortage and is altered by parasitism	Increased movement may be associated with aggression and jockeying for position as refuge shortages increase. We were uncertain about specifying the nature of an interactive effect of parasitism. Debilitating effects of infection might reduce movement rates, and so might diminish the increase in movement with crowding. Alternately, increased movement with crowding might be exacerbated by parasitism if infected gobies were forced to cover larger areas than uninfected ones.
5	Feeding rates are not affected by parasitism or refuge shortage	Parasite-mediated TMIIs do not involve competition for food, so feeding rates should be unrelated to refuge shortage and parasitism

## Results

### Prediction 1: The rate of aggressive interactions increases with refuge shortage and the increase is amplified by parasitism

**Figure 2:**
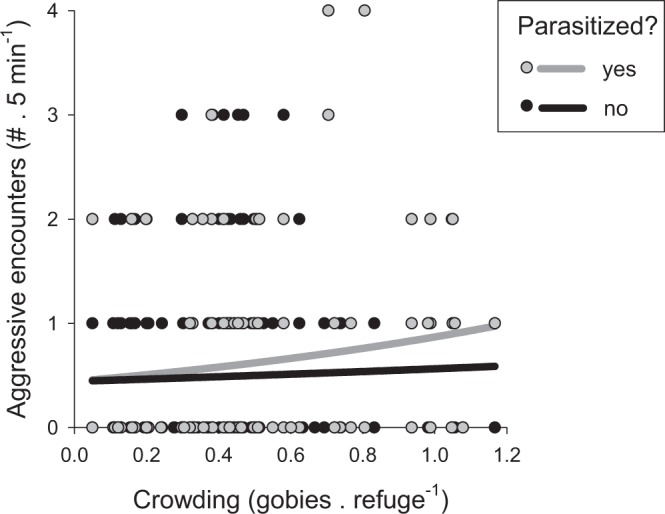
Parasitism increases the effect of crowding on aggressive interactions. The rate of aggressive encounters is plotted as a function of a measure of crowding that measures the shortage of refuges from predation (the ratio of gobies to refuges). Data are plotted separately for parasitized fish (grey symbols and regression line, n = 157) and unparasitized fish (black symbols and regression line, n = 424). Many points overlap and so are not visible. Regressions lines show the significant parasitism × crowding interaction term estimated using a generalized linear model that specified a negative binomial distribution and a log-link function (see Methods for details).

Focal individuals were observed for 5-minute periods (n = 581 focal observations), and we recorded the number of aggressive interactions (chases) involving conspecifics (n = 313 encounters in total). Gobies experiencing higher levels of refuge shortage had more aggressive interactions than those in areas where refuges were in greater supply, and this increase in aggressive encounters was experienced more strongly by parasitized gobies than uninfected ones (Generalized linear model: parasitism × refuge shortage interaction term, Wald χ^2^ = 4.80, df = 1, p = 0.028). These results thus provide support for prediction 1 (Fig. 2).

### Prediction 2: The likelihood of losing an aggressive encounter increases with refuge shortage and the increase is amplified by parasitism

**Figure 3:**
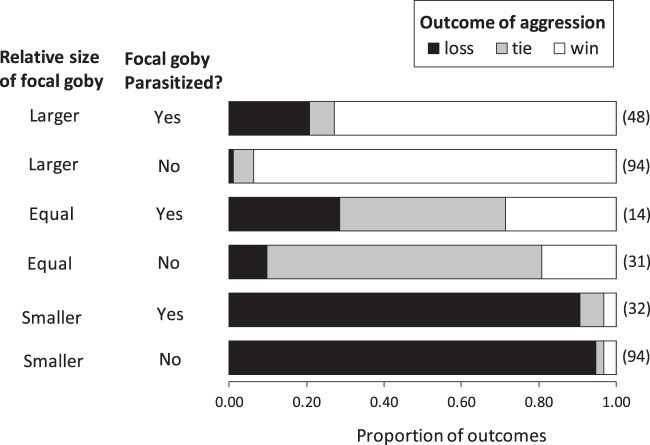
The outcome of aggressive interactions with conspecifics is affected by relative size and parasitism. Relative size refers to whether the focal goby was smaller, larger, or visually indistinguishable in body size from the conspecific with which it was interacting. Outcomes are classified as wins (focal goby chased the other goby), losses (focal goby was chased), or ties (no clear winner). Data plotted are the proportion of outcomes grouped by relative size and parasitism and the number of observations is shown in brackets to the right of each bar.

We also classified the outcome of each interaction with a conspecific as a win (focal goby chased the other goby), a loss (focal goby was chased), or a tie (no clear winner). Because relative size strongly influences the outcome of interference competition in many species, with larger-bodied individuals typically outcompeting smaller ones^22,23^, we also visually estimated the body length of the focal goby (larger, smaller, or too close to distinguish visually) relative to its counterpart in each aggressive encounter. Relative size was a strong predictor of winning or losing during an aggressive encounter (Fig. 3). When focal observations of all gobies were pooled, whether parasitized or not, focal individuals won almost all encounters when they were larger than the other goby (n = 126 encounters, 94% = wins). Correspondingly, focal gobies almost always lost encounters when they were smaller than the other goby (n = 142 encounters, 87% = losses). When the two interacting fish were similar in size, the outcome was less clear-cut (n = 45 encounters, 62% = ties, 16% = losses, 22% = wins).

Because of the strong advantage conferred by larger size, we tested whether the degree of size-advantage was modified by parasitism (Fig. 3). To do this, we tabulated interactions where the outcome was unexpected based on relative size. We classified these unexpected outcomes as either positive (winning an encounter with a larger fish) or negative (losing an encounter with a smaller fish). The frequency of unexpected positive outcomes was unaffected by parasitism (Chi^2^ contingency test: df = 2, χ^2^ = 1.32, p = 0.52), with both infected fish and uninfected fish occasionally winning encounters with a larger fish (infected fish: n = 94 encounters, 3% = wins; uninfected fish: n = 126 encounters, 3% = wins). Parasitism did, however, increase a focal goby’s likelihood of losing an encounter despite having a size-advantage (Chi^2^ contingency test: df = 2, χ^2^ = 17.6, p = 0.0006). Unparasitized gobies lost only 1% of encounters when they were larger than the other goby (n = 94 encounters), whereas parasitized fish lost 21% of encounters when they were larger (n = 142 encounters) (Fig. 3).

Prediction 2 would be supported if the rate of unexpected losses increased with refuge shortage more rapidly for infected gobies than for those without parasites. Because unexpected losses were rare (n = 11 encounters in 2900 minutes of focal observation), the data were too sparse to test this prediction (Generalized linear model: parasitism × refuge shortage interaction term could not be estimated). Consequently, although it was clear that unexpected losses were more likely for infected gobies than uninfected ones, we could not test whether these losses were more frequent when refuges were in short supply and so we lacked sufficient evidence to conclusively evaluate prediction 2.

### Prediction 3: Distance to refuges declines with increased refuge shortage and the decline is lessened by parasitism

**Figure 4:**
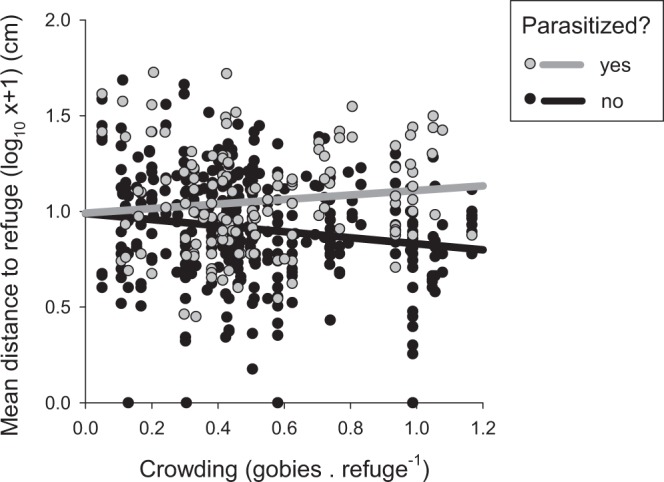
Parasitism increases the effect of crowding on proximity to refuges. The mean distance from a refuge (estimated every 30 seconds during a 5-minute observation period) is plotted as a function of crowding that measures the shortage of refuges from predation (the ratio of gobies to refuges). Data are plotted separately for parasitized fish (grey symbols and regression line, n = 157) and unparasitized fish (black symbols and regression line, n = 424). Regression lines show the significant parasitism × crowding interaction term estimated with an ANCOVA model using log_10_(x + 1) transformed data (see Methods for details).

Because gobies flee to refuges only when threatened or attacked, we visually estimated their distance from a potential refuge every 30 seconds during each 5-minute focal observation. As the shortage of refuges intensified, uninfected gobies spent progressively more time closer to a refuge (Fig. 4). Parasitized gobies, in contrast, were generally further from refuges and they displayed no tendency to be closer to potential shelter as refuges became scarce (Fig. 4). This parasite-mediated change in behaviour was statistically significant (Linear model: parasitism × refuge shortage interaction term, F_1,576_ = 8.77 p = 0.003), providing support for prediction 3.

### Prediction 4: Movement increases with increased refuge shortage and the increase is altered by parasitism

**Figure 5:**
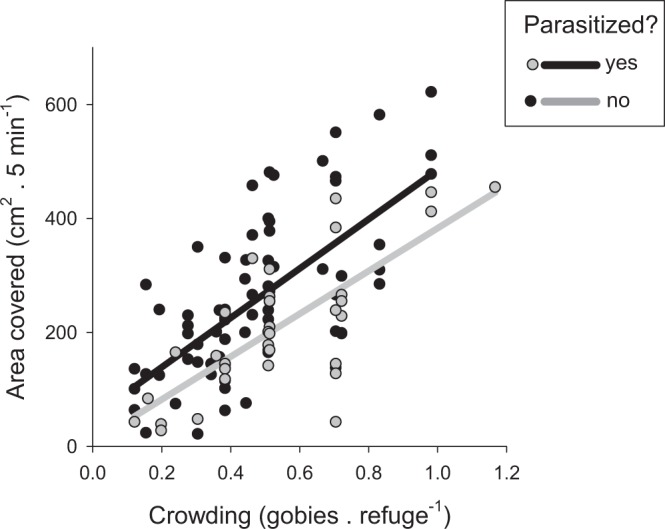
Crowding and parasitism influence movement rates. Movement (area covered during a 5-minute observation period in cm^2^) is plotted as a function of crowding that measures the shortage of refuges from predation (the ratio of gobies to refuges). Data are plotted separately for parasitized fish (grey symbols and regression line, n = 33) and unparasitized fish (black symbols and regression line, n = 74). Regression lines for parasitized and unparasitized fish were fit using a linear model and differ significantly in elevation but not in slope (see Methods for details).

To quantify movement, we tracked the location of a subset of focal gobies (n = 107 focal observations) on a scaled map of the area to estimate the total area covered in 5 min. Gobies spent most of their time still on the bottom and made intermittent movements that often appeared to be associated with feeding or interacting with other individuals. Gobies covered progressively larger areas as the shortage of refuges intensified (Linear model: main effect of refuge shortage, F_1,103_ = 81.8, p < 0.0001). At all levels of refuge shortage, parasitized gobies covered smaller areas than those free of infection (Linear model: main effect of parasitism, F_1,103_ = 0.80, p = 0.37), but there was no evidence of an interactive effect of refuge shortage and parasitism (Linear model: parasitism × refuge shortage interaction term, F_1,103_ = 0.41, p = 0.53) (Fig. 5). Increased movement rates were thus associated with competition for refuges, but there was no parasite-mediated effect of crowding on movement that would provide support for prediction 4.

### Prediction 5: Feeding rates are not affected by parasitism or refuge shortage

We quantified focal gobies’ feeding rates (bites minute^−1^) by recording the number of bites during each focal observation. Infected gobies fed at lower rates than uninfected ones (means ± SE: parasitized gobies = 0.57 ± 0.05; unparasitized gobies = 0.75 ± 0.03) (Linear model: main effect of parasitism, F_1,578_ = 3.70, p = 0.002). Feeding rates were, however, unaffected by refuge shortage (Linear model: main effect of refuge shortage term, F_1,578_ = 0.024, p = 0.87) and nor was there an interactive effect of refuge shortage and parasitism (Linear model: parasitism × refuge shortage interaction term, F_1,577_ = 0.25, p = 0.62). The results are thus consistent with prediction 5 that this parasite-mediated TMII does not involve competition for food.

## Methods

### Study system

The host species, the bridled goby, is a small benthic fish that is abundant on most Caribbean coral reefs. After a pelagic larval stage, gobies settle to reef habitats at 6.5–8 mm standard length (SL). Gobies are short-lived (most <1 year), and reach a maximum size of 50–55 mm SL. They are sedentary after settlement and occupy small home-ranges (<2 m^2^ in area). Home ranges include both sand and reef, because gobies feed mostly on sand-dwelling invertebrates but use crevices at the junction of reef and sand as refuge from predation^19^. We compiled observations from the two habitat types where bridled gobies are common: (1) small patch reefs surrounded by sand, where gobies reside at the sand/reef interface, and (2) larger continuous expanses of habitat where live or dead coral is interspersed with enough sandy areas for feeding (Table 1).

Predation on gobies is most often inflicted by several species of larger piscivorous fishes^17^. Gobies are cryptic on sand and sometimes remain motionless when predators approach. More often, however, they flee to a refuge when approached or attacked (81% of escapes observed in nature, n = 21; and 87.5% of unpublished lab trials, n = 16). Goby home ranges usually contain more than one refuge and home ranges often overlap. As local density increases and a goby’s home range overlaps with more neighbours, competition for refuges arises as gobies vie for access to refuges when predators approach.

The parasitic copepod (*P. tortugensis*) that infects the gill cavity of bridled gobies is reported to infect several other fish species^24^. However, at our study site infections were only detected in bridled gobies and two ecologically similar congeners of *C. glaucofraenum* (*C. dicrus* and *C. eidolon*)^16^. Laboratory and field experiments confirm that the parasite has a direct life cycle and is transmitted directly between neighboring gobies^16^. After a free-swimming naupliar stage that allows newly hatched individuals to infect a new host, copepods lose their swimming ability and cannot subsequently switch hosts^16,24^. Adult male and female copepods attach themselves to the branchial chamber and gills of gobies and their presence is associated with mucus production plus damage to the gill cavity and respiratory surfaces^16^. Similar signs of damage have been observed in a few uninfected gobies (0.6%, *n* = 505), suggesting that gobies can shed infections, but that shedding infection is rare^16^. Parasitic infections have been observed on fish from 9.7–44.4 mm SL (*n* = 505), but because most parasitic infections (85%) occurred on fish from 14–26 mm SL^16^ we compiled behavioural observations of parasitized gobies spanning this size-range (Supplementary Table S1). Observations were taken at sites near Guana Island, British Virgin Islands (64° 35 121′W, 18° 29′N). The prevalence of the parasite was low when we first began studying the gobies at this site (prevalence = 2.7% from 1992–4, *n* = 220) but reached 32.4% in 2004 (*n* = 310) and has remained between 20–27% since then.

### Study design

We compiled behavioural observations from four studies (Table 1). The design and execution of study 1 has been reported previously^19^ and is therefore summarized only briefly. Using a cross-factored design, gobies were transplanted among small experimental plots to create a different density in each plot (Supplementary Table S2). Coral/rubble was added to half of the plots to increase the number of refuges, and divers then counted the final number of refuges to ensure that plots differed in refuge density (Supplementary Table S2, Supplementary Fig. S1). Study 3 has not been previously reported but was a repeat of study 1 and so was done in the same plots and used almost identical treatments and methods (Supplementary Table S2, Supplementary Fig. S1).

The design and execution of study 2 has also been reported previously^15^. In this observational study, gobies were observed within a large (560 m^2^) expanse of continuous goby habitat that was subdivided into a 2 × 2 m lattice with nails hammered into the substratum as markers. Using these markers, twenty 4 × 4 m study plots were designated within the site. Goby density and refuge density within each plot was then quantified by divers and focal gobies were subsequently observed within each plot.

Study 4 has not been previously reported, but it’s design and execution closely follow previous manipulations of goby density on small patch reefs at the same study site^25–27^. Study 4 used 12 patch reefs that were constructed from pieces of coral rubble (Supplementary Fig. S1) in a sandy bay. Each patch reef was separated by 10 m to negate goby movement among reefs. The patch reefs were similar in size and construction and so varied little in refuge density (Supplementary Table S2). A different number of gobies was transplanted to each reef to create a gradient of goby density (Supplementary Table S3) but, unlike previous manipulations, we deliberately transplanted both infected and uninfected gobies to the reefs in varying proportions (Supplementary Table S3). Because most of the gobies used in studies 1–3 were at the larger end of the infection-prone size-range, we used mostly gobies from the smaller end of the infected size-range for study 4 (Supplementary Table S1).

### Observations of focal gobies

At intervals during each experiment, observations were made on the behavior of gobies on each study plot or reef (Table 1). Most observations lasted 5 minutes (*n* = 557) but in some cases we lost track of the goby earlier (*n* = 24). Focal individuals were haphazardly selected as encountered, so the number of observations of infected gobies (n = 163, 28% of the total) and uninfected gobies (n = 418, 72% of the total) roughly matched the prevalence of infected and uninfected fish in the population (20–27%). Each focal goby was carefully approached by a diver, who remained still roughly 1.5–2 m from the goby during the observation period to minimize the chance of influencing the goby’s behaviour (Supplementary Fig. S2). We visually diagnosed the focal goby as infected or uninfected by copepods, which can be done with reasonable accuracy (90% accuracy, *n* = 187) because infected gobies usually have a distinctive distended operculum (Supplementary Fig. S3)^21^. Most errors in diagnosis are false negatives because gobies infected with just one or two juvenile copepods do not have distended opercula. We also visually estimated the body length of focal gobies and classified their size relative to gobies with whom they interacted aggressively (larger, smaller, too close to distinguish visually). By catching and measuring gobies after some observations (*n* = 43), we found visual estimates of body length to be accurate within 3 mm SL and fish classified as visually indistinguishable differed by ≤2 mm SL. When gobies were tagged (studies 1 and 3), we could recognize gobies as individuals and tried to avoid making repeated observations of the same fish. It is likely that some repeat observations occurred in studies 2 and 4, but because these repeats were probably uncommon, we treated focal observations as independent replicates in the analysis.

The work reported here was performed in accordance with relevant guidelines and approved by the University of Rhode Island Institutional Animal Care and Use Committee (Protocols AN01-08-003, AN02-08-003 and AN02-09-005).

### Statistical analysis

We tested our hypotheses using linear models that included terms for parasitism (a fixed categorical variable: levels = infected and uninfected), refuge shortage (a continuous variable), and the interaction term (parasitism × refuge shortage). The test of the interaction term was of the most importance ecologically, because a significant effect was interpreted as providing support for a parasite-mediated TMII. Models also included a term for study (a random categorical variable: levels = 1, 2, 3 and 4), to account for any other differences between the four studies from which data were compiled. Because differences between studies were always small and non-significant, we present pooled data in the results.

Data on movement (area covered) conformed to the assumptions of linear models and were analyzed using an analysis of covariance (ANCOVA) model. We tested the assumption of homescedasticity within each treatment group by inspecting plots of studentized residuals against predicted values for each group and using Levene’s test for equality of variances. We tested for normality by inspecting normal Q-Q plots and using the Shapiro-Wilk test for normality. Data on feeding rates and distance to shelter also met the assumptions of linear models after log transformation to equalize variances, and so transformed data were analysed using ANCOVA models. Data on aggressive encounters were moderately zero-inflated and overdispersed counts. We therefore fitted different generalized linear models (GLMs) appropriate for count data with this structure (Poisson distribution with log link function, negative binomial distribution with log-link function, zero-inflated negative binomial model) and selected the best fitting model (negative binomial distribution with log-link function) using information theoretic criteria^28^. We confirmed that the best-fitting model conformed to assumptions of GLMs by inspecting plots of residuals against predicted values and normal Q-Q plots.

When analysing the outcome of aggressive encounters, we expected that relative body size (whether the focal goby was large, smaller, or similar in size to its counterpart) would have a strong influence on the outcome^15,21^. We therefore first grouped interactions by relative size, and for each group created a two-way contingency of outcome (wins, losses and ties) by parasitism (infected or uninfected). We used χ^2^ tests of independence to test the null hypothesis that the frequency of outcomes was unaffected by parasitism.

## Discussion

### Goby behaviours are consistent with parasite-mediated competition for refuges

There were interactive effects of refuge shortage and parasitism for two of the four behaviours we predicted might be associated with parasite-mediated competition for refuges: the rate of aggression and proximity to refuges. We manipulated refuge shortage and so provide strong evidence that refuge shortage caused these changes in behavior. To be equally confident that changes in host behavior were caused by parasites, we should have experimentally infected a random sample of hosts and compared them to uninfected controls^29^. Because we simply correlated parasite presence with host responses, we cannot exclude the alternative possibility that inherent differences in goby aggression and activity influence susceptibility to infection. To alter our conclusions about a TMII, however, these inherent differences between gobies would have to influence not just their susceptibility to infection, aggression and movement, but also how they change with refuge shortage – which we consider unlikely.

Past work showed that gobies engage in aggressive encounters with conspecifics more often as refuge shortage increases^19,21^. We showed here that the increase was more pronounced for infected gobies than uninfected ones and so is consistent with a TMII (prediction 1). We also predicted that a synergistic effect of parasitism and refuge shortage might influence the outcome of aggressive interactions but, although parasitism increases the likelihood of losing an aggressive encounter, we found no evidence that this increased probability of losing varied with refuge shortage (prediction 2). Because gobies may flee to more than one crevice when threatened and refuges are not obviously guarded, the link between aggression and access to refuges is not as straightforward for gobies as it is for species who defend access to a single refuge^30^. Nonetheless, gobies have spatial memory that allows them to learn the location of refuges^31^ and, like many species, fleeing towards a refuge overrides other considerations that affect the directionality of escape^32–34^. We therefore hypothesize that, regardless of the outcome of aggression, an increased frequency of aggressive encounters may affect mortality risk by compromising awareness of approaching predators, the location of potential refuges, or both.

Gobies’ proximity to refuges was also consistent with a parasite-mediated TMI. Uninfected gobies tended to be closer to potential shelter as refuge shortage increased, but infected gobies were generally further from shelter and did not move closer to shelter as refuges became scarce (prediction 3). Past work in which the two factors were tested separately using fewer observations detected no effects of parasitism^15^ or refuge shortage^19^ on gobies’ proximity to shelter. We suggest that testing both factors together using a larger sample size facilitated the detection of an interactive effect in the present study. We also predicted that the area covered, while foraging might be influenced by an interactive effect of parasitism and refuge shortage (prediction 4) but, although the area covered by gobies was reduced by parasitism and increased by refuge shortage, these effects were independent rather than interactive. Although the movement of gobies is not linked to a TMII, proximity to refuges is plausibly related to parasite-mediated competition for refuges based on the assumption that spending more time close to a refuge increases the chance of escape when attacked. There is little direct evidence to support this assumption, because behavioural studies quantifying escape responses are rarely performed with real predators^35,36^. Indirect support for this assumption, however, is provided by the common finding that fish reduce the distance at which they react to predators when closer to a refuge, which implies a reduced perception of risk^37^.

### Goby behaviours are not consistent with parasite-mediated competition for food

Animals infected with parasites may either increase or decrease foraging activity, depending on the energy drain associated with infection and other specifics of the host-parasite infection^10,38^. We found reduced feeding rates in parasitized gobies, for which there are several possible explanations. First, gobies mostly feed by winnowing invertebrates from the sand, and copepod infection causes damage to the gills and branchial chamber that may disrupt sorting of food and non-food items^16^. Second, based on other changes in behaviour (increased gill ventilation rates, reduced area covered) and morphology (reduced gonad mass and somatic growth) in parasitized gobies^15,16^, infection may simply be debilitating enough to result in lower activity^10^.

Parasite-mediated competition for food is possible because infection can impair the capture efficiency of hosts that hunt evasive prey, which should reduce the host’s ability to compete for food^39,40^. Some of the major components of the sand-dwelling meiofauna on which gobies feed (harpacticoid copepods, ostracods, and polychaetes) are demersal zooplankton that enter the water-column at night. We cannot eliminate the possibility of food-limitation without manipulating the food supply but, because their prey are redistributed every night, it is doubtful that gobies can deplete their local food supply. This, coupled with the fact that goby feeding rates are density-independent^41^ suggests that, although feeding rates were reduced in parasitized gobies, gobies are unlikely to compete for food. For species that use refuges for safety but must leave refuge to feed, there is often a trade-off between foraging and predator avoidance^42^. This creates the possibility for TMIIs based on parasite-induced behaviours that mediate the trade-off between foraging and refuge use^43,44^, but the weight of circumstantial evidence suggests that this TMII does not involve competition for food (prediction 5).

### Similarities to, and differences from other parasite mediated TMIIs

The interaction between bridled gobies, copepods and predators (Fig. 1) falls within a general class of TMIIs, in which parasites modify a predator-prey interaction^6,8^. Most such interactions identified to date involve trophically transmitted parasites and are interpreted as manipulations of host behaviour that benefit the parasite. Most frequently, parasite-induced changes to the behaviour of intermediate hosts enhance predation by the final host and so facilitate parasite transmission^12,13^. In some cases, however, parasite-mediated host-manipulation increases predation on infected intermediate hosts by additional predators as well as the final host which may diminish the benefit to the parasite^45^. Alternately, when the parasite it is not sufficiently mature to infect the predator, it is also possible for behavioural manipulation to reduce predation by the final host because trophic transmission would not benefit the parasite^46–48^. The short-term effect of host-manipulation can thus create TMIIs that either enhance or diminish predation, and the long-term consequences may be complex^45^. Host manipulation is also possible for directly transmitted ectoparasites, like *P. tortugensis* infecting bridled gobies, and is predicted to favour predator avoidance behaviours by the host, because death of the host also means death for the parasite. We are aware of just one potential example consistent with this prediction, in which parasitized mosquito larvae were less active and spent more time in refuges than uninfected larvae, and consequently suffered less predation^49^. For bridled gobies, parasitism enhanced rather than reduced the impact of predation and so the parasite-induced behaviours are not consistent with host-manipulation.

We hypothesize that the altered behaviours we observed in parasitized gobies are not the result of host manipulation but may instead be coincidental to infection^13^. In other words, they may simply be a side-effect of compromise to sensory, neurological or physiological systems due to infection by *P. tortugensis*. Circumstantial evidence supporting this conclusion comes from the impacts of other ectoparasitic copepods and isopods that infect the gills of their fish hosts. Like *P. tortugensis*, these parasites can be large relative to the host, feed on blood and cause damage to the gills and branchial chamber. Infected fish show various debilitating symptoms, including depression of the heart and pericardial cavity, reduced respiratory metabolism, chronic inflammation and increased mucus production that may lead to neoplasia^50–53^.

The TMII involving gobies, copepods, and predatory fishes may, therefore, be grouped with other TMIIs in which apparently coincidental side-effects of infection increase the susceptibility of hosts to predation. In most such cases, the parasite-induced changes on host phenotype are morphological. For example, polychaete infestation weakens whelk shells and so increases their vulnerability to predation by shell-crushing crabs^54^. Similarly, predatory fish selectively consume *Daphnia* infected with bacteria because infected *Daphnia* are more opaque than uninfected ones and are more easily detected^55^. Lastly, nematode infection damages the caecal mucosa of grouse so that they emit more scent than uninfected birds and, as a result, they are more easily detected by mammalian predators^56^. The interaction between gobies, copepods, and predatory fishes differs from these other TMIIs in the mechanism by which parasitism enhances host-susceptibility to predation. Although the long-term consequences of TMIIs are difficult to predict^57,58^, we suggest that one distinctive feature of this TMII worth exploring is that predation is strongly density-dependent. Because parasitism enhances the density-dependent component of mortality, it may thus have a stabilizing effect on goby abundance.

## Supplementary information

Supplemental information

## Data Availability

The datasets analysed during the current study are available from the corresponding author on reasonable request.
