# Supplementary material for: Behavioural mechanisms underlying parasite-mediated competition for refuges in a coral reef fish
Source: Sci Rep. 2019 Oct 29;9:15487. doi: 10.1038/s41598-019-52005-y (PMC6820773; doi:10.1038/s41598-019-52005-y)
Supplement: Supplementary file 1 — Supplemental information [file 41598_2019_52005_MOESM1_ESM.pdf]

Supplemental information

Behavioural mechanisms underlying parasite-mediated competition for refuges in a coral reef fish

Graham E. Forrester, Erin Chille, Katie Nickles, and Kiran Reed

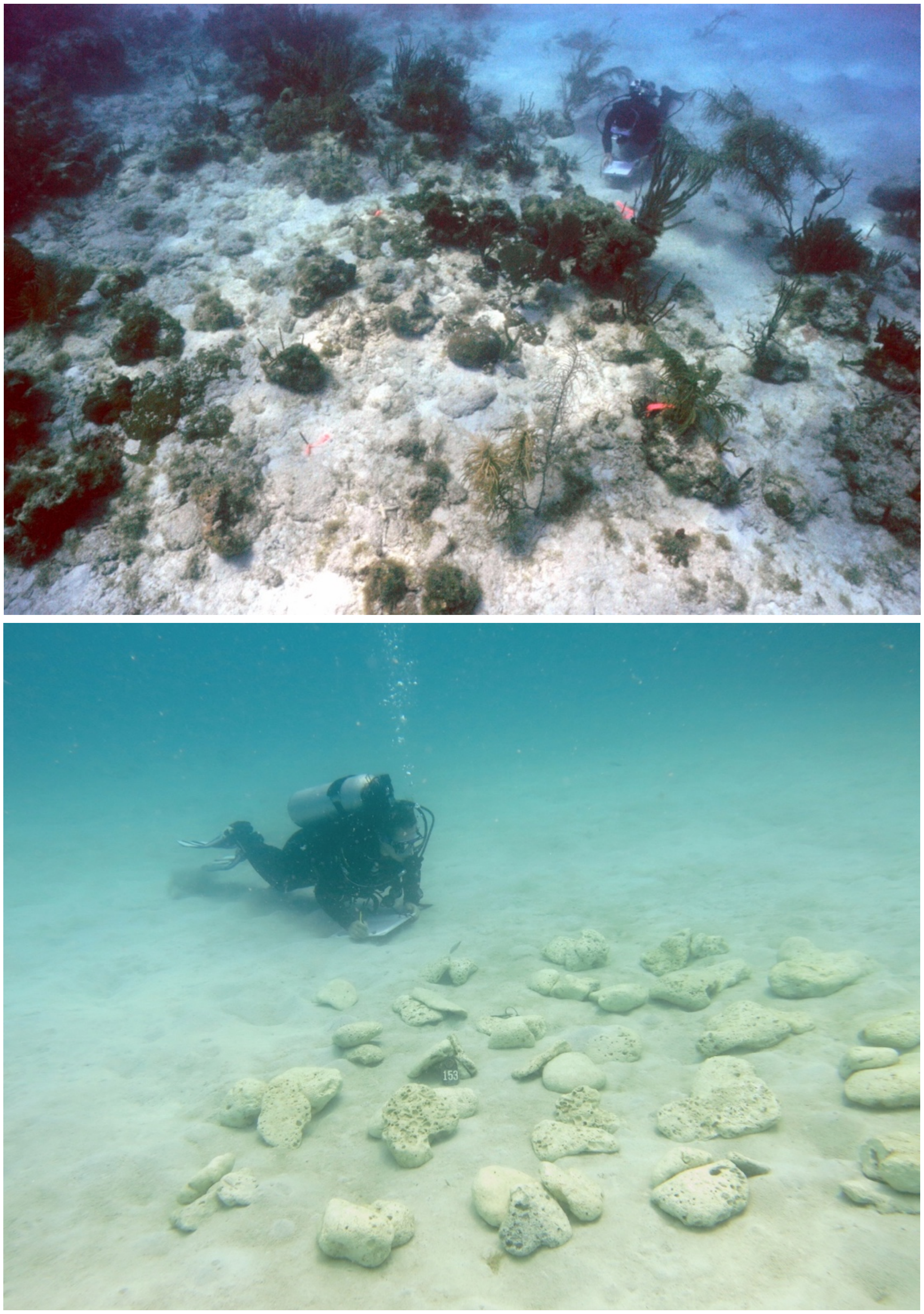

Supplementary Figure S1. Photographs of experimental replicates. Top picture - one of the authors (GF) observing gobies in the 4 x 4 m plots within continuous expanse of goby habitat used in 2000, 2001 and 2016. Bottom picture - one of the authors (KR) observing gobies on 2 x 2 m experimental patch reefs used in 2018. Bottom picture by GF, top picture by Rachel Finley - used with permission.

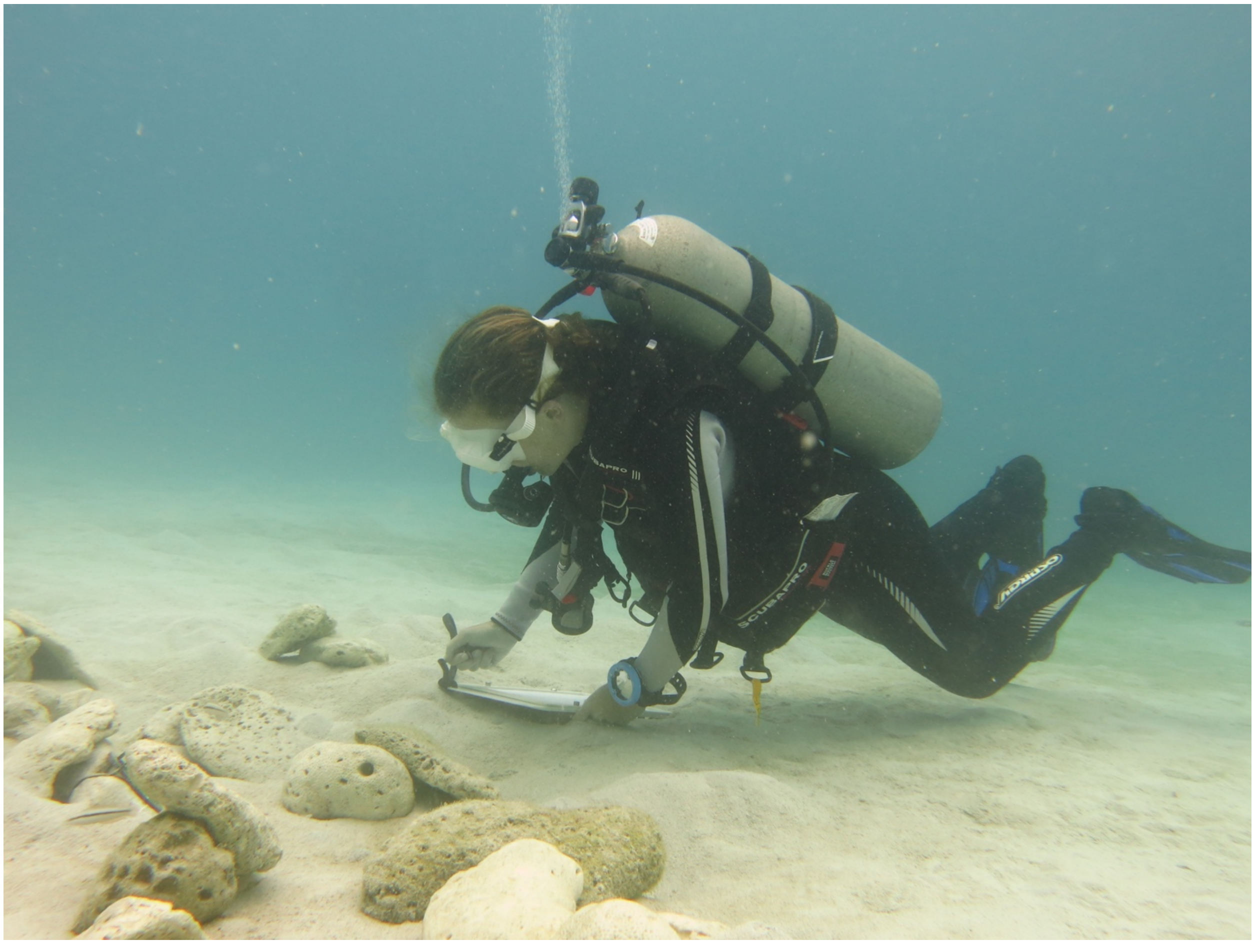

Supplementary Figure S2. One of the authors (KR) observing a focal goby on one of the 2018 patch reefs. Picture by GF.

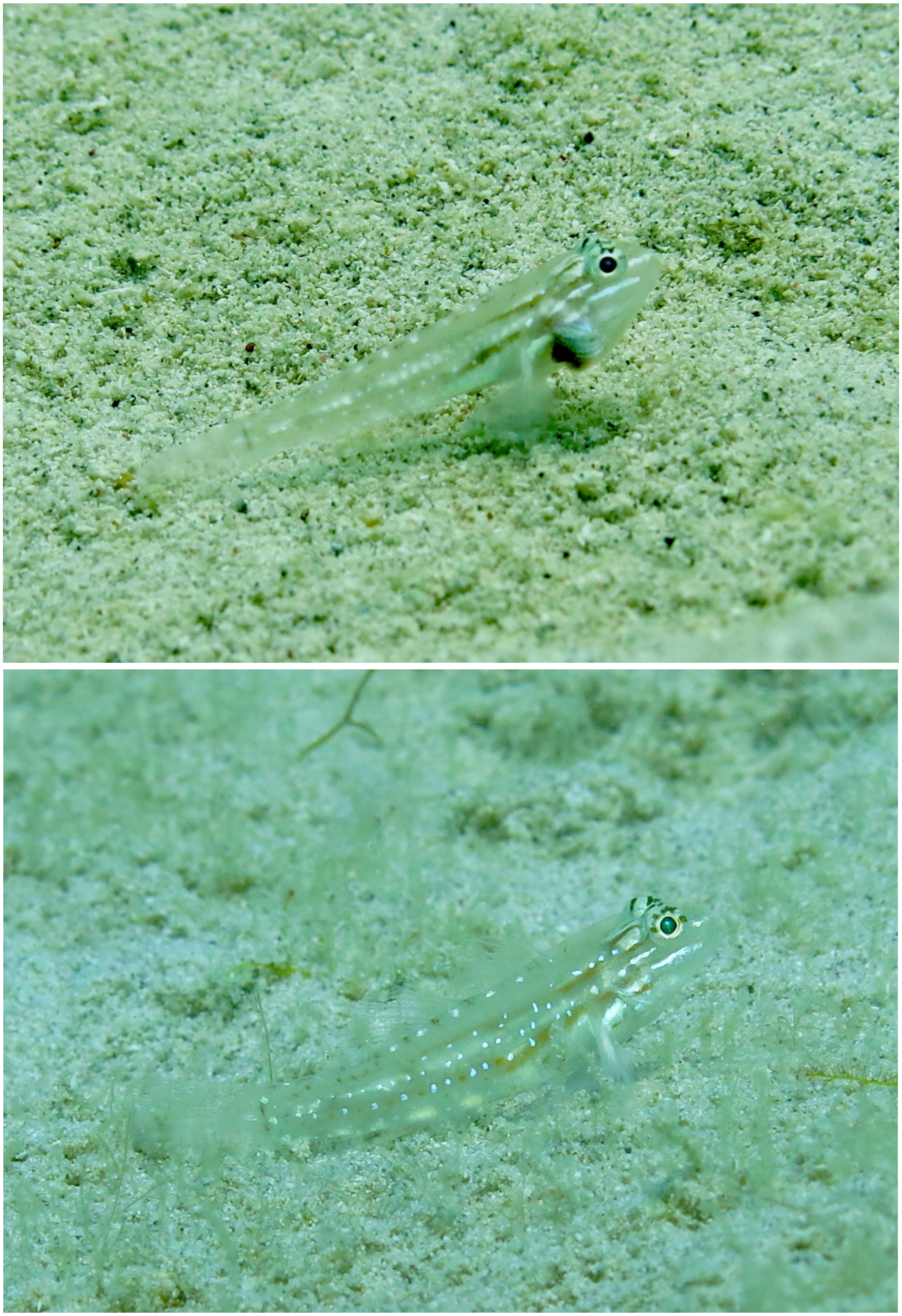

Supplementary Figure S3. Top picture - A parasitized goby with the distended operculum used to diagnose infection. Lower picture - an uninfected goby. Pictures by GF.

Study	Year	Body length (mm SL)			
		Mean	SE	Min.	Max.
1	2000	27.8	4.2	22	45
2	2001	25.8	4.9	17	40
3	2016	29.7	5.7	21	43
4	2018	17.0	3.8	15	30

Supplementary Table S1. Summary of goby body sizes in the four studies from which behavioural observations of focal gobies were compiled. Displayed are the mean, standard deviation (SE), and the minimum (Min.) and maximum (Max.) for each study.

Study	Year	Goby density (# m^{-2})				Refuge density (# m^{-2})				Refuge shortage (gobies per refuge)			
		Mean	SE	Min.	Max.	Mean	SE	Min.	Max.	Mean	SE	Min.	Max.
1	2000	1.8	1.2	0.2	4.8	4.6	2.1	1.5	9.7	0.47	0.33	0.05	1.08
2	2001	1.7	0.5	0.5	2.7	4.6	2.0	1.9	9.4	0.42	0.23	0.12	1.05
3	2016	2.1	1.3	0.5	4.7	4.9	2.2	1.8	9.6	0.47	0.27	0.12	0.98
4	2018	6.6	4.5	0.6	15.2	12.5	6.1	0.9	19.6	0.54	0.25	0.11	1.17

Supplementary Table S2. Summary of levels of goby density, refuge density, and refuge shortage for the four studies from which behavioural observations of focal gobies were compiled. In all four studies, the goby and refuge density differed in each study plot. Displayed are the mean, standard deviation (SE), and the minimum (Min.) and maximum (Max.) for each study.

Reef	Infected hosts		Uninfected hosts	
	# reef^{-1}	# m^{-2}	# reef^{-1}	# m^{-2}
1	0	0.0	9	2.9
2	0	0.0	18	5.8
3	0	0.0	28	9.0
4	1	0.3	35	11.3
5	2	0.6	4	1.3
6	5	1.6	28	9.0
7	6	1.9	20	6.5
8	7	2.3	27	8.7
9	10	3.2	9	2.9
10	10	3.2	11	3.5
11	12	3.9	30	9.7
12	13	4.2	34	11.0

Supplementary Table S3. Response surface design of study 4, a field experiment testing the relative effects of the densities of infected and uninfected gobies. Displayed are the starting number and density of gobies on each patch reef. Based on visual inspection, infected hosts were infected with at least one large female copepod, and so likely to be infectious, whereas uninfected hosts had no visible symptoms of infection.
